# Extending Effective Dynamic Range of Hyperspectral Line Cameras for Short Wave Infrared Imaging

**DOI:** 10.3390/s22051817

**Published:** 2022-02-25

**Authors:** Muhammad Saad Shaikh, Keyvan Jaferzadeh, Benny Thörnberg

**Affiliations:** 1 Department of Electronics Design, Mid Sweden University, Holmgatan 10, 85170 Sundsvall, Sweden; benny.thornberg@miun.se; 2 Department of Computer Science and Software Engineering, Concordia University, Montreal, QC H3G 1M8, Canada; keyvan.jaferzadeh@mail.concordia.ca

**Keywords:** hyperspectral imaging, push-broom camera, waste sorting, calibration, teflon, PTFE, dark current, InGaAs, plastic detection, polymer classification

## Abstract

In this work, a multi-exposure method is proposed to increase the dynamic range (DR) of hyperspectral imaging using an InGaAs-based short-wave infrared (SWIR) hyperspectral line camera. Spectral signatures of materials were captured for scenarios in which the DR of a scene was greater than the DR of a line camera. To demonstrate the problem and test the proposed multi-exposure method, plastic detection in food waste and polymer sorting were chosen as the test application cases. The DR of the hyperspectral camera and the test samples were calculated experimentally. A multi-exposure method is proposed to create high-dynamic-range (HDR) images of food waste and plastic samples. Using the proposed method, the DR of SWIR imaging was increased from 43 dB to 73 dB, with the lowest allowable signal-to-noise ratio (SNR) set to 20 dB. Principal Component Analysis (PCA) was performed on both HDR and non-HDR image data from each test case to prepare the training and testing data sets. Finally, two support vector machine (SVM) classifiers were trained for each test case to compare the classification performance of the proposed multi-exposure HDR method against the single-exposure non-HDR method. The HDR method was found to outperform the non-HDR method in both test cases, with the classification accuracies of 98% and 90% respectively, for the food waste classification, and with 95% and 35% for the polymer classification.

## 1. Introduction

Spectral imaging allows spectral information to be acquired across the electromagnetic spectrum [1]. While an ordinary camera can only capture light in the visible spectrum, spectral imaging can capture the infrared region of the EM spectrum, visible spectrum, ultraviolet, and x-rays, as well as different combinations of the four. Spectral imaging is divided into different categories depending upon the number of spectral bands used, the width of the spectral bands and the gaps between them, and the spectral resolution. These abstractly defined categories are known as Multi-Spectral Imaging (MSI) and Hyper-Spectral Imaging (HSI). MSI generally collects spectral data from up to ten different generally noncontiguous bands, while HSI collects spectral data from hundreds of bands with a high spectral resolution. The use of spectral imaging was first proposed in 1985 by Goetz [2] for remote sensing of Earth. Currently, spectral imaging is being used in many different areas of scientific research and engineering applications. Satellite-based remote sensing [3], agriculture [4], the defense industry [5], medical diagnostics [6], and food inspection [7] are just a few examples of situations in which the use of spectral imaging is very popular. Diffusely reflected EM waves contain specific object signatures depending upon the temperature and material of the reflecting surfaces in the scene [8]. In other applications, reflected signatures are captured by a camera and then fed into a Machine Learning (ML) algorithm to detect and classify the objects present in the scene [9].

**Figure 1:**
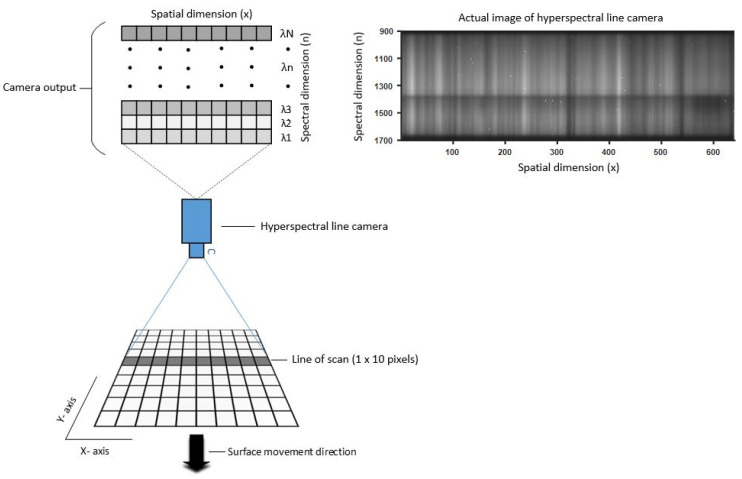
Depiction of the hyperspectral camera in use and the output of the camera.

A common problem with imaging technology is that optical sensors have a much lower dynamic range (DR) than real-world scenes [10]. Theoretically, the DR of a camera is defined as the ratio between the highest and lowest intensities that the camera can record. However, it is practically impossible to use the whole DR as per the definition–the low-intensity values have a very poor signal-to-noise ratio (SNR). It is necessary to define a minimum acceptable SNR for individual applications; this is known as ‘foot-room’ [11]. A common approach to solving this problem is using multiple exposures of the same scene. This multi-exposure approach was first developed in the late 80s [12,13] and early 90s [14]. Since then, the multi-exposure method has been widely used to increase the DR of imaging to produce more visually realistic images [15,16,17,18,19]. Previous studies report the use of the multi-exposure method in visible spectrum imaging where CCD or CMOS silicon-based photodetectors are used. For short-wave infrared (SWIR) imaging, indium gallium arsenide (InGaAs) sensors are used. InGaAs cameras typically have spectral ranges from 900 to 1700 nm but can be sensitive up to 2500 nm. InGaAs photodetectors have their own drawbacks that mean they are generally incapable of high-precision low-light imaging. These limitations are ‘read noise’ and ‘dark current’ (DC). Read noise is the fundamental uncertainty in measurement, and DC is the exposure time and temperature-dependent thermal phenomena generated by the spontaneous movements of electrons. InGaAs-based cameras use Peltier cooling modules to minimize DC; however, the order of magnitude of the noise sources in InGaAs sensors is much higher than the usual levels for silicon-based cameras. As exposure time increases, DC increases linearly and occupies more of the remaining DR of the SWIR camera. Figure 1 depicts how a hyperspectral line camera reads a signal from a surface or material. For each imaging shot, the camera reads a scan line and sends the data to a PC. To scan a whole surface, either the camera or the object being scanned (here along the *y*-axis) must be able to move.

In this article, we argue that the widely known multi-exposure approach can be adapted for InGaAs-based hyperspectral line cameras to increase the DR of hyperspectral SWIR imaging. The aim is to improve the SNR of low-intensity measurements in the presence of DC while avoiding saturation at high intensities. Unlike existing multi-exposure fusion algorithms, the proposed algorithm incorporates DC modeling, which cannot be ignored for InGaAs-based sensors. We also include a criterion for measurements to have a minimum SNR. This criterion defines the foot-room for all measurements. Any signal below the foot-room has an SNR that is too low to be considered reliable. This foot-room is non-usable space in the DR. The usable DR beyond the foot-room and up to the saturation level is referred to in this paper as ‘effective DR’.

In the next section, we present the materials and methods used in this work, followed by the results and discussion. The paper ends with a conclusion and list of references.

## 2. Materials and Methods

In this section, we present brief details about the hardware used in this research and the methods used to acquire and process the data. We also present a formalization of the multi-exposure method to extend the DR of hyperspectral imaging.

### 2.1. Hyperspectral Camera

**Figure 2:**
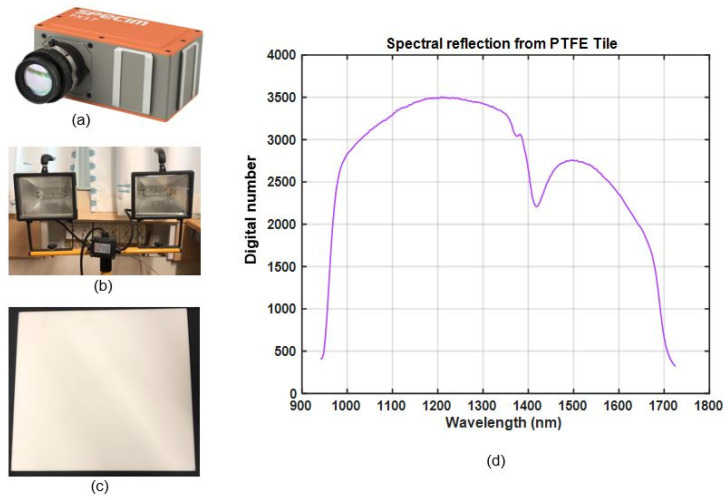
Materials used in the research: (**a**) Specim FX17e hyperspectral camera, (**b**) two halogen lamps mounted on a tripod, (**c**) PTFE tile, (**d**) average intensity of spectra for the PTFE tile.

To measure the spectral reflection of test samples, we used the Fx17e hyperspectral camera from Specim, as shown in Figure 2a. The camera operates in the SWIR region, with an exact range of 900–1700 nm, and can capture 224 spectral bands with a full width half maximum (FWHM) of 8nm. This camera uses the push-broom method [20] to collect spectral information of the surface along the line of scan. To scan a complete surface, either the camera or the surface must be moved perpendicular to the line of scan (see Figure 1).

### 2.2. Illumination Source

To illuminate the test samples and reference target we used two halogen lamps (Anslut 420,083 IP44) mounted on a tripod stand, as shown in Figure 2b. The lamps were connected to a general-purpose power outlet of 220 V. We assumed the supply voltage was constant throughout the experiments.

### 2.3. Reference Target

To measure the relative spectral reflectance of test samples, we needed a reference target material. We choose a 10 mm thick, 295 × 295 mm polytetrafluoroethylene (PTFE) tile manufactured by a Swiss company, Amsler & Frey. This board is shown in Figure 2c and was used as the reference for all the experiments presented in this paper. This PTFE tile is a low-cost alternative to the conventionally used material Spectralon^TM^. It is known to diffusely reflect light with as even a distribution as Spectralon^TM^ over the range of all wavelengths in near-infrared (NIR) regions [21]. This PTFE tile is also insensitive to humidity, a property of particular interest when experimenting in harsh environments like waste management facilities. Figure 2d shows the average spectra of light reflected by the reference target.

### 2.4. Experiment Setup

**Figure 3:**
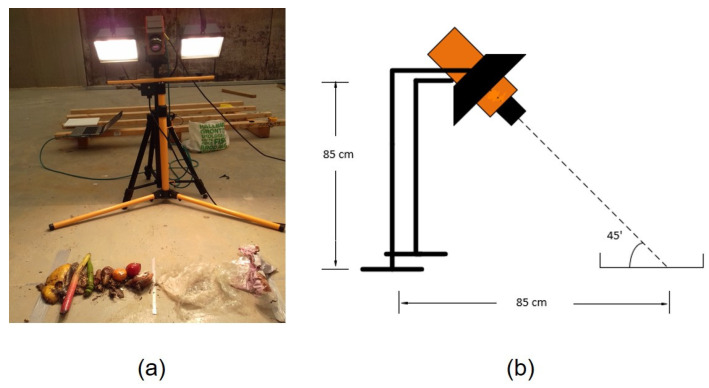
Experiment setup: (**a**) photo of the experiment setup, (**b**) schematic depiction of the experiment setup.

**Figure 4:**
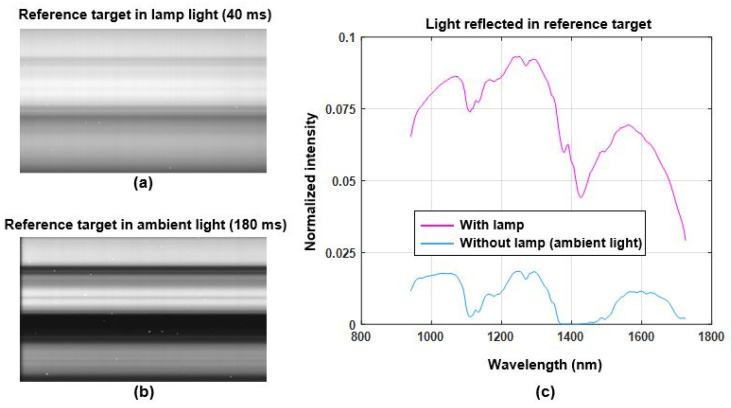
Light profile during experiments at HEMAB: (**a**) reflection from reference target when illuminated by halogen lamps at 40 ms exposure time, (**b**) reflection from reference target in ambient light at 180 ms exposure time, (**c**) normalized average intensity spectra of (**a**,**b**).

All measurements of the test samples and reference target were taken using the same experiment setup, shown in Figure 3a,b. The camera was placed between two halogen lamps at a height of 85 cm, with all three facing downward at an angle of approximately 45 degrees relative to the ground plane. This experiment setup was designed to avoid specular reflections as much as possible while still providing a strong enough signal from the diffused reflections. All experiments for the first test case, plastic detection in food waste, were conducted inside the waste storage hall of the HEMAB [22] gasification plant. Ambient light entering through the opaque ceiling and reflections from walls and other objects in close proximity to the experiments may have added a small amount of background bias to the measurements. However, the only dominant source of illumination at the time of the experiments was the halogen lamps included in the experiment setup. Figure 4 shows hyperspectral measurements of the reference target when illuminated by the halogen lamps (Figure 4a; exposure time is 40 ms) and in ambient light (Figure 4b; exposure time is 180 ms). Figure 4c shows the normalized average intensity of spectra for the reference target presented in Figure 4a,b. Both graphs in Figure 4c are normalized such that the measurements are independent of the exposure time and DC. The normalization process is explained in greater detail in Section 2.8. For the second test application case of polymer classification, all the experiments were conducted inside the vision lab at the department of electronics, Mid Sweden University using similar experiment setup shown in Figure 3.

### 2.5. Test Cases

#### 2.5.1. Case 1: Detection of Plastic in Food Waste

The detection of plastic in food waste was chosen as a test application for this study. Many studies have proposed the use of spectral imaging to detect and classify plastic in waste [23,24,25]. It is important to separate plastic from food waste to prevent it from being trapped in transportation channels when it is moved to gasification tanks, which stops the entire process. The sample food waste was composed of a variety of objects with very different reflectance properties in SWIR. It also included unwanted plastics from food packaging, grocery bags, etc. This combination of food waste and plastics results in a very high DR for the scene.

#### 2.5.2. Case 2: Polymer Classification

To test the applicability and robustness of the proposed method we chose polymer classification as the second test case. Polymer classification is needed to sort out different types of polymers before recycling. We chose polymer classification as the second test case as different types and colors of polymers can create a scene of high DR.

### 2.6. Sample Preparations

#### 2.6.1. Test Case 1

**Figure 5:**
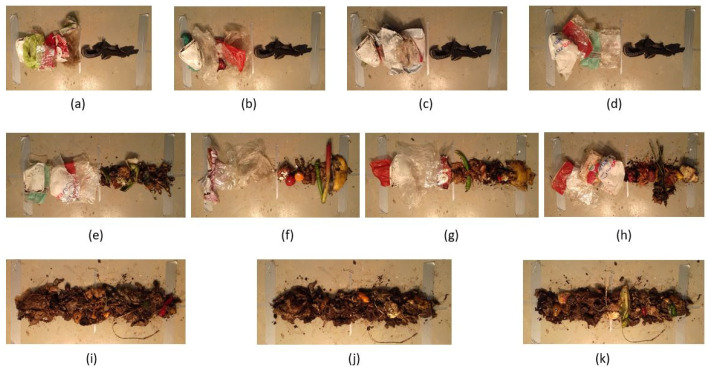
(**a**–**d**) show samples of general plastic (left) and black plastic (right). (**e**–**h**) show the samples of general plastic (left) and food (right). (**i**–**k**) show the samples of decomposed food.

The sampling site for test case 1 is located at the storage hall of the HEMAB [22] gasification plant. Food waste is delivered to the company on trucks and unloaded in the storage hall for the gasification and food fertilization processes. The waste is mainly composed of food leftovers from households. The plant receives food waste in recyclable paper bags and it usually contains different types of unwanted plastics that most likely come from food packaging and grocery bags. To prepare the samples, the plastics were first carefully hand-picked and placed inside the camera’s field of view (FOV). Plastics of different colors, materials, and conditions were picked to increase the variety in the samples. Having variety in the sample space is particularly important for the training of ML algorithms and classification results. For the food waste, half of the samples were composed of comparatively undecomposed food items. These items were hand-picked from the waste and placed in the imaging area. The other half of the food samples was composed of comparatively decomposed food items, from which it was impossible to identify individual food items; these food samples also included biodegradable paper bags. During the preparation and imaging of the samples, special attention was paid to black plastics. Black plastics were treated as a separate class of material and imaged and sampled separately from other plastics as they are known for being difficult to detect due to their low reflectivity [26]. Before placing materials for imaging, we marked the imaging area with duct tape. The thick duct tape at the sides of the samples defined the camera line edges of the scan, and the thin duct tape defined the line of scan and divided the camera FOV in half. These markings were helpful for the preparation of samples and, particularly, the labelling of data. Figure 5 shows all eleven (a–k) samples used in this study. Most of the samples were prepared with two different class items to increase the DR of the samples. A description of each sample is presented in Table 1.

#### 2.6.2. Test Case 2

The test samples for case 2 were composed of six different types of recyclable plastics and black plastics (also recyclable). These recyclable plastics are categorized by their recycling numbers from 1 to 7. We used the first 6 types in this test case. Table 2 presents the details of used plastics with their polymer names, recycling numbers and abbreviations.

The black plastic used in this test case belonged to type 5, but treated as a separate class. In total 29 samples were prepared and imaged to have sufficient number of training and testing vectors from each plastic type. In Table 3 below, the description of each sample is presented.

To test proposed method, it was necessary for samples to have DR more than the effective DR of the camera. For that purpose, samples were prepared carefully with all types of polymers including the low reflecting black plastic. The DR of all samples combined was 59 dB. See Section 2.8 for the computation of DR.

### 2.7. Dark Current

**Figure 6:**
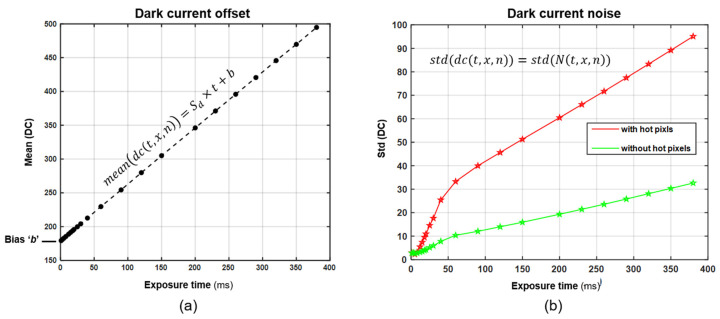
DC: (**a**) Mean DC vs. Exposure time, (**b**) Standard deviation of DC.

DC is the flow of electrons when no photons are present in the photodetectors. It is a thermal phenomenon generated by the spontaneous movement of electrons within the photodetectors (valence electrons move to the conduction band by thermal excitation) [27]. The amount of pixel DC is directly proportional to the exposure time. However, the amount of DC varies from pixel to pixel. DC can be modelled as follows:

(1) $$dc\left(t,x,n\right)=S_{d}\times t+N\left(t,x,n\right)+b$$

where ‘$S_{d}\times t$’ represents the DC offset value for the entire image for an exposure time ‘*t*’, ‘*N*(*t*, *x*, *n*)’ is a zero-mean map of noise, and ‘*b*’ represents the bias; the pixels are indexed by ‘*x*’ and ‘*n*’. Figure 6 shows the relationship between the mean DC and standard deviation of the DC (random noise) and the exposure time of the hyperspectral camera used. We measured the mean DC values at different exposure times and found that they increase linearly with exposure time. Estimating DC is particularly important for calculating camera DR and SNR, as it is one of the major factors that reduces the DR of InGaAs SWIR camera sensors [28]. Although InGaAs detectors provide sensitivity over an extended SWIR range due to their lower bandgap, they result in a much higher DC compared to silicon-based detectors. InGaAs cameras require very deep cooling (i.e., down to −85 °C) to minimize this unwanted noise source. Any signal below the DC noise floor is not meaningful. A detailed DC estimation model for the Specim Fx17e hyperspectral camera is presented in [21].

### 2.8. Dynamic Range of an Analyzed Surface

**Figure 7:**
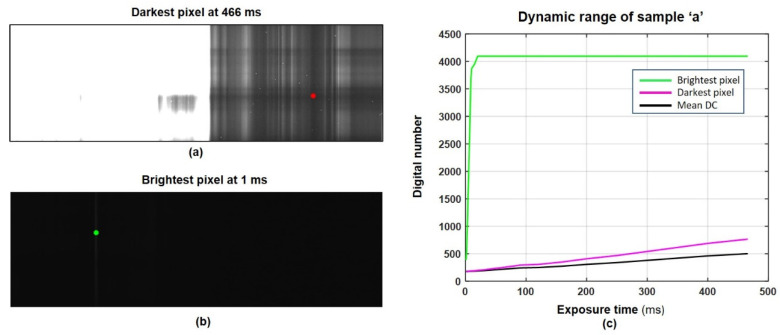
Spectral reflection at minimum and maximum exposure times of sample ‘a’: (**a**) darkest pixel in the image at 466 ms exposure time, (**b**) brightest pixel in the image at 1 ms exposure time, (**c**) raw values of the brightest and darkest pixel, and mean DC values in sample images at different exposure times.

In this subsection, the DR of sample ‘a’ is calculated experimentally. Sample ‘a’ was selected because it has the highest DR of all the samples shown in Figure 5. Figure 7 shows two images taken at the gasification plant of sample ‘a’ for exposure times of 1 ms and 466 ms respectively. At an exposure time of 1 ms, the camera captures a very weak signal where even the brightest pixel in the image only has a value of 385 on a 12-bit scale. As per Figure 7c, the brightest pixel of the dark image approaches saturation at an exposure time around 10 ms, but we chose to capture the dark image at 1 ms to provide the maximum saturation margin for measuring even brighter pixels. For the image in Figure 7, taken with an exposure time of 460 ms, a large number of camera pixels hit the saturation level and therefore the image does not contain any meaningful information. Meanwhile, the darkest pixel in the image measures just above the noise floor with a very low value of 765 on a 12-bit scale.

Figure 7c shows the camera’s raw output values for the brightest and darkest pixels and mean DC values with respect to the exposure time. These raw values represent the combined dose of energy from incident photons, DC charges, sensor bias, and random noise. If ‘*P*’ represents the set of pixels (image) indexed by ‘*x*’ and ‘*n*’ at exposure time ‘*t*’ and $S_{d}+S_{p}$ represents the combined intensity (slope) of DC and absorbed photons:

(2) $$P\left(t,x,n\right)=b+N\left(t,x,n\right)+\left(S_{d}+S_{p}\right)\times t$$

To measure the incident intensity of light, we normalized the measured pixel *P* such that the light intensities were independent of exposure time and DC:

(3) $$I\left(x,n\right)=\frac{P\left(t,x,n\right)–\;mean\left(dc\left(t,x,n\right)\right)\;}{t}$$

The DR of the surface ‘*R_S_*’ is calculated as the ratio between the normalized intensities of the brightest pixel and the darkest pixel. If ‘*I_B_*’ represents the brightest intensity, and ‘*I_D_*’ represents the darkest intensity, we can calculate the DR of the scene as:

(4) $$R_{S}=20\cdot log\;\left(\frac{I_{B}}{I_{D}}\right)\approx 56\;\mathrm{dB}$$

The scene formed by the sample ‘a’ has a DR of 56 dB, which implies that capturing the given scene with a single exposure requires a camera with a minimum DR of 56 dB.

### 2.9. Dynamic Range of a Camera

The DR of a camera is ideally defined as the ratio between the highest possible intensity value that the camera can record without saturation and the minimum intensity values above the noise floor. DR defines the operating range of a camera within which meaningful data can be extracted. It is generally presented in decibels (dB) using the following equation:

(5) $$R=20\cdot log\;\left(\frac{Max\;signal\;intensity}{Min\;signal\;intensity}\right)$$

**Figure 8:**
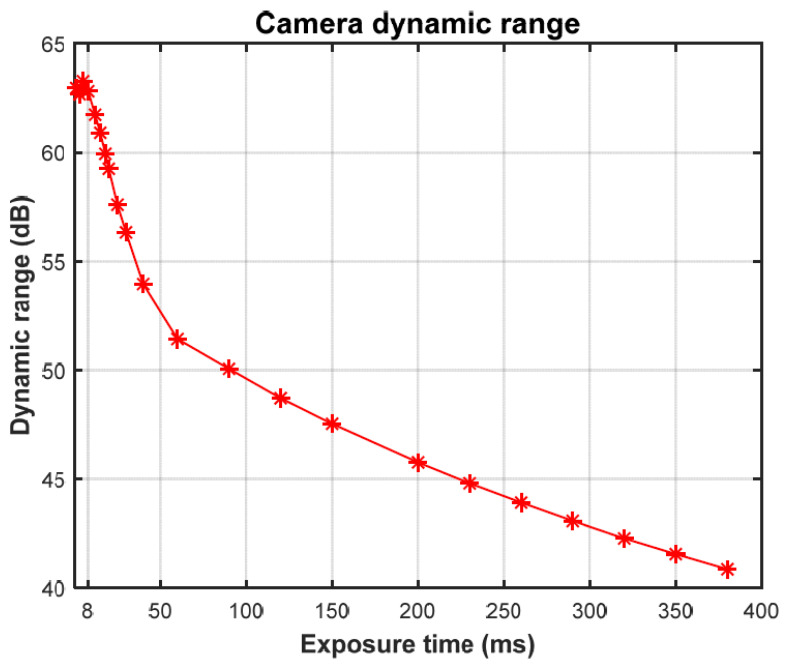
Camera DR vs. exposure time.

In the case of the spectral camera used in this study, for which the major source of noise is DC, the above equation is rewritten to calculate the camera’s DR ‘*R_C_*’ [29]:

(6) $$R_{C}=20\cdot log\;\left(\frac{M-mean\left(\mathrm{DC}\right)}{std\left(\mathrm{DC}\right)}\right)$$

where *M* is the maximum digital value (2^12^ − 1), the upper limit of the hardware’s capacity to measure signal intensity. The DC should be measured prior to imaging the samples and then subtracted from the acquired hyperspectral images [30]. A random small variation in DC from pixel to pixel, estimated by the standard deviation of DC, is taken as the minimum signal intensity measurable by the camera. All spectral measurements used in this work were taken in 12-bit grayscale format. To calculate the DR of the camera at different exposure times, we calculated the DC values and standard deviation of DC from Figure 6. Figure 8 shows the calculated DR of the hyperspectral camera ‘*R_C_*’ at different exposure times. The DR of the camera is inversely proportional to exposure time, while the DC offset is directly proportional to exposure time.

As per Figure 8, the camera has a DR ‘*R_C_*’ greater than ‘*R_S_*’ (56 dB), but only for exposure times less than approximately 30 ms. As per Figure 7c, the intensity values of the darkest pixel in the scene for exposure times less than 30 ms are almost equal to the DC. This implies that the lower intensity pixels in a given image have an SNR almost equal to 1 (0 dB) and do not contain any meaningful information. To measure the meaningful spectral reflection of sample ‘a’, we set a minimum SNR ‘*minSNR*’ criterion. A signal with an SNR lower than the *minSNR* will be considered unmeaningful. For example, a *minSNR* of 20 dB will reduce the DR of the spectral camera showed in Figure 8 by 20 dB and make it impossible to capture the scene of the given sample ‘a’. Considering the *minSNR* criterion, Equation (6) for the effective DR ‘*R_E_*’ is rewritten as follows:

(7) $$R_{E}=20\cdot log\left(\frac{M-mean\left(DC\right)}{std\left(DC\right)\cdot 10^{\frac{minSNR}{20}}}\right)$$

That is equal to:

(8) $$R_{E}=R_{C}-minSNR$$

Using a camera with the highest DR at the shortest exposure time will put extremely high demands on the power and intensity of the illumination source, an impractical and costly requirement for many users and applications. It is necessary to have a multi-exposure method that measures the reflections from highly reflective surfaces with a short exposure time and low reflective surfaces with a long exposure time. In the next subsection, we present the multi-exposure method used to collect the spectral information of waste samples using the Specim Fx17 SWIR hyperspectral line camera.

### 2.10. Multi-Exposure Method

**Figure 9:**
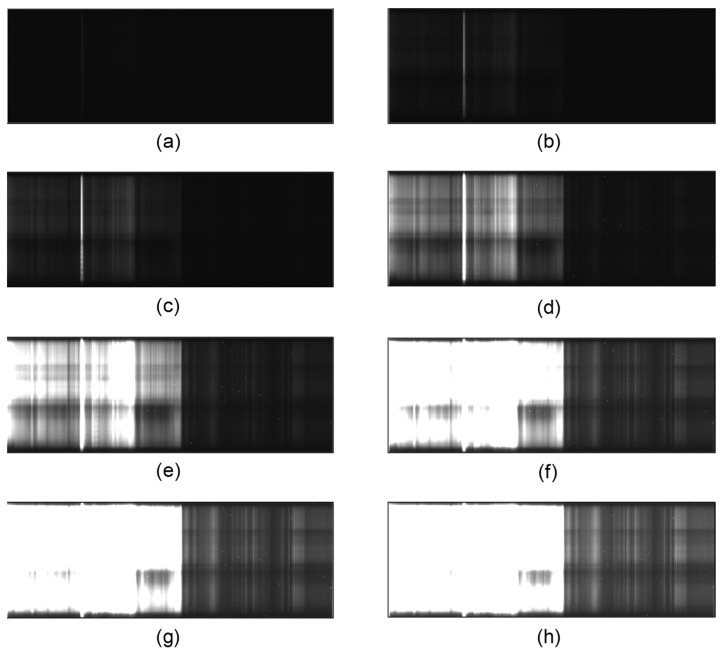
Sample ‘a’ imaged at different exposure times (ms): (**a**) 1 (**b**) 5 (**c**) 10 (**d**) 50 (**e**) 120 (**f**) 250 (**g**) 350 (**h**) 466.

We can increase the exposure time to capture larger doses from relatively unreflective materials, but that will cause the doses from highly reflective materials in the same scene to saturate the camera detector. An HDR image of each sample was obtained by processing the series of respective sample images taken at different exposure times. Each sample was illuminated with the halogen lamp and images were captured using a set of exposure times: T = {1, 2, 3, 4, 5, 6, 7, 8, 9, 10, 15, 20, 30, 40, 50, 60, 70, 80, 90, 120, 160, 200, 250, 300, 350, 400, 466} ms. Some of these images are shown in Figure 9. The reference target was also imaged with an exposure time of 40 ms.

To construct an HDR image of the waste sample, we created an empty image and inspected the images pixel by pixel in descending order of exposure time. If a pixel was near saturation, we looked for the same pixel in the next image taken with a shorter exposure time. We repeated this process until we found the unsaturated (with a margin of 20 digital numbers) pixel. Once the pixel was found from the stack of images, we wrote the value for the corresponding pixel in the HDR image as per the following equation:

(9) $$I_{\mathrm{HDR}}\left(x,n\right)=\frac{\sum_{i=1}^{k}\;P\left(t_{i},x,n\right)-mean\left[DC\left(t_{i},x,n\right)\right]}{\sum_{i=1}^{k}{\;t}_{i}}$$

$P\left(t_{k},x,n\right)$’ represents the first image where the unsaturated pixel is found and ‘$t_{i\;}\in T$’ represents the exposure time of the image ‘$P\left(t_{i},x,n\right)$’. This procedure is applied to every single pixel of the image. The computed image ‘$I_{\mathrm{HDR}}\left(x,n\right)$’ uses the same concept of light intensity as previously described for a single exposure by Equation (3). Using this multi-exposure method, we calculated the extended effective DR ‘*R_EM_*’. The maximum possible signal strength that can be captured by this camera is M (2^12^ − 1) minus the mean DC for a given minimum exposure time. The minimum recordable signal is just above the noise floor at a given maximum exposure time. Let ‘$t_{s}$’ be the minimum exposure time and ‘$t_{m}$’ be the maximum exposure time; we can calculate the extended DR ‘*R_EM_*’ as follows:

(10) $$R_{EM}=20\cdot log\left(\frac{\left[M-mean\;\left(DC\left(t_{s}\right)\right)\right]\cdot \;t_{m}}{std\left(DC(t_{m}\right))\;\cdot \;t_{s}}\right)\;–\;minSNR$$

Let us assume the minimum signal strength must be at least ten times stronger than the noise to be considered meaningful (*minSNR* = 20 dB). Calculating the values of DC offset and DC standard deviation from Figure 6, we get *R_EM_* ≈ 73 dB. Since the DR of sample ‘a’ was 56 dB, there is a saturation margin of 17 dB, while the SNR is never lower than 20 dB for any of the pixels.

### 2.11. Spectral Calibration

The sensitivity of the spectral bands of the camera used in this research was considered to be unknown. The spectral distribution of light intensity for the halogen lamps was also unknown. Therefore, a calibration procedure was needed to measure the relative reflectance of the samples independently of different combinations of line spectral cameras and light sources. The relative reflectance is defined as the wavelength-dependent fraction of reflected light intensity from the analyzed surface versus the reflected light intensity from the reference target. The exact spectral calibration procedure we applied to measure the relative reflectance of food and plastic samples is presented in [21].

### 2.12. Data Preparation

#### 2.12.1. Test Case 1

The data used in test case 1 was all based on the eleven samples shown in Figure 5. For each sample, a corresponding HDR image was computed using Equation (9). Once the HDR image was computed, the relative reflectance of the scene was computed with respect to the reference target and stored in a 2D matrix. Each column of the relative reflectance matrix was labelled as one of the three classes: food, plastic, or black plastic. It was made sure that the duct tape and small floor section between the two materials were not labelled as any data class. For that, forty columns at the center of all the images were ignored and not used for labelling, training of support vector machine (SVM) classifiers, or classifying test data. For example, for sample ‘a’ the first 300 columns were labelled as ‘bright plastic’, then 40 columns were ignored, and columns 341 to 640 were labelled as ‘black plastic’. All eleven samples were processed in the same way to extract HDR data vectors (columns of HDR matrix) and labelled by their respective classes.

For the preparation of non-HDR data, single images with 6 ms exposure times were selected from each sample. Images with 8 ms exposure times were the brightest for all sample images without any pixel exceeding the saturation level. However, to provide a margin of saturation, we selected images with 6 ms exposure times. Images with 6 ms exposure times provide 3 dB of saturation margin for the recorded samples. The relative reflectance of each sample was measured. As with the HDR data, 40 columns at the center of each matrix of relative reflectance were ignored and the rest of the columns labelled by their respective classes. For all HDR and non-HDR data, 3120 food, 2400 plastic, and 1200 black plastic data vectors were available. Approximately 80% of the data vectors were used for training the SVM classifiers and 20% were randomly selected as test data. Table 4 shows the number of training and test vectors used to perform classification.

#### 2.12.2. Test Case 2

Data used in test case 2 was based on 29 samples described in Table 3. For every sample a corresponding HDR image was computed using Equation (9). Once the HDR image was computed, the relative reflectance of the scene was computed with respect to the reference target and stored in a 2D matrix. Each column of the relative reflectance matrix was labelled as one of the six types of recyclable plastics or black plastic. For the preparation of non-HDR data, single images of 3 ms exposure time were selected from each sample as some of the samples’ images were hitting saturation when exposure time was 4 ms. Then the relative reflectance of each sample was measured using selected images. Every column of non-HDR relative reflectance matrix was also labelled as a corresponding class of plastics. This pre-processing of data resulted in equal number of data vectors for same classes in HDR and non-HDR datasets. From every HDR and non-HDR class, approximately 90% randomly selected data vectors were reserved for the training of SVM classifiers and 10% were reserved for testing. Below in Table 5, number of data vectors per each class of plastics are mentioned.

### 2.13. Principal Component Analysis

Principal Component Analysis (PCA) is a mathematical method for dimensionality reduction that is often used for large data sets; it transforms a large set of variables into a smaller one but still preserves the trends in the original data set. The principal components (PC) of a data set are a sequence of direction vectors that best fit the data while being orthogonal to other vectors. A best-fitting vector is defined as one that minimizes the average squared distance between the data points and the vector itself. These vectors constitute an alternate orthonormal basis for the same data set in which different individual dimensions are linearly uncorrelated. PCA is frequently used for band selection in the field of hyperspectral imaging [9,31]. It is also used to reduce dimensions to visualize hyperspectral images and develop classification algorithms for applications with high dimensional input data sets.

**Figure 10:**
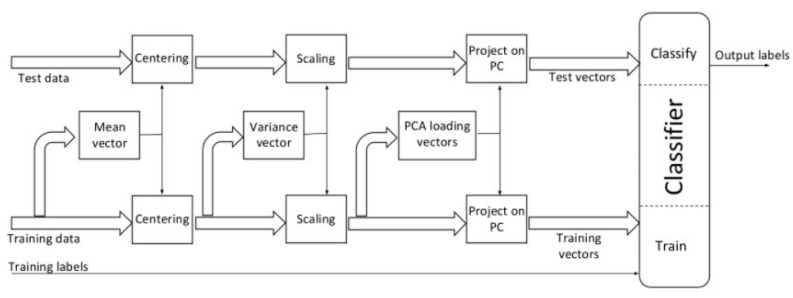
Pre-processing steps of training and test data sets.

For each test case, we performed two separate PCAs on training sets of the HDR and non-HDR data vectors respectively to visualize perspective plots of data classes in three-dimensional space and create low dimensional training sets. As shown in Figure 10, the centering and scaling of test datasets were conducted using mean and variance vectors of training data vectors, respectively. The PC from the respective PCAs were used to transform the HDR and non-HDR test vectors into a lower-dimensional space as with the training data. PCA was applied to the training sets only; the same pre-processing and projection of test vectors into the same lower-dimensional space ensured that the test vectors had no impact on the training of the classifier and vice versa.

### 2.14. Support Vector Machines

Support vector machines (SVMs) are supervised learning models with associated learning algorithms that are used for data classification and regression analysis. In this study, we trained two SVM classifiers to demonstrate that the proposed multi-exposure method not only increases the DR of hyperspectral imaging but also results in better classification of materials in scenes. To implement multiclass SVM we used MATLAB multiclass error-correcting codes (ECOC) [32] with radial base function (RBF) kernels. We used the combination of PCA for dimension reduction and SVM for classification as the previous study [33] reported that this combination is computationally inexpensive and results in better classification of hyperspectral data.

## 3. Results and Analysis

**Figure 11:**
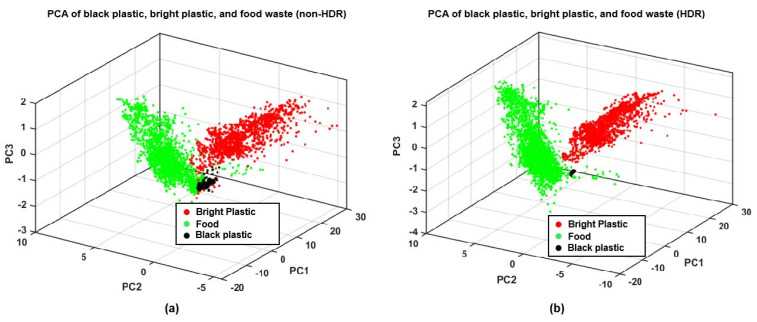
PCA plots for food and plastic: (**a**) non-HDR, (**b**) HDR.

For test case 1, PCA of food and plastic measurements was performed for both HDR and non-HDR data. For the PCA shown in Figure 11, the black plastic and bright plastic classes were treated separately for visualizing data clusters. The plastic collected from the waste for sampling had small traces of decomposed food, and the food samples may also have had some impurities, so 5% of outlying data were removed from all three classes. Figure 11a shows the PCA of the non-HDR data, and Figure 11b shows the PCA of the HDR data.

For the training and testing of classifiers, both plastic classes were treated as a single plastic class. However, we kept track of the plastic classes to later analyze which is more prone to be wrongly classified as food waste. Approximately 80% of the randomly mixed data vectors for all three classes from the PCAs shown in Figure 11 were used to train two SVM classifiers–one for non-HDR data and one for HDR data. Approximately 20% of the randomly mixed data vectors were used to test the classifiers.

**Figure 12:**
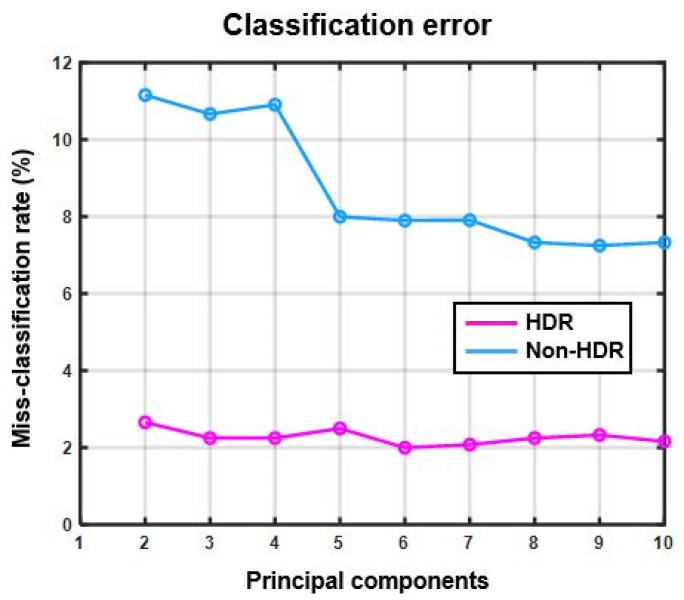
Miss-classification rate (%) vs. number of principal components (test case 1).

Figure 12 shows the miss-classification rate of SVM classifiers for both non-HDR and HDR data with respect to the increasing number of principal components added as inputs to the classifiers.

Figure 12 shows the percentage of plastic wrongly classified as food waste or vice versa, but it does not provide any information on how much the black or bright plastics contribute to the overall error rate individually. In Table 6, confusion matrices are presented to show the results of classifiers for non-HDR and HDR data with three, four, and five principal components. In the confusion matrices, it is possible to see the exact number of black or bright plastics that were wrongly classified as food waste and vice versa.

**Figure 13:**
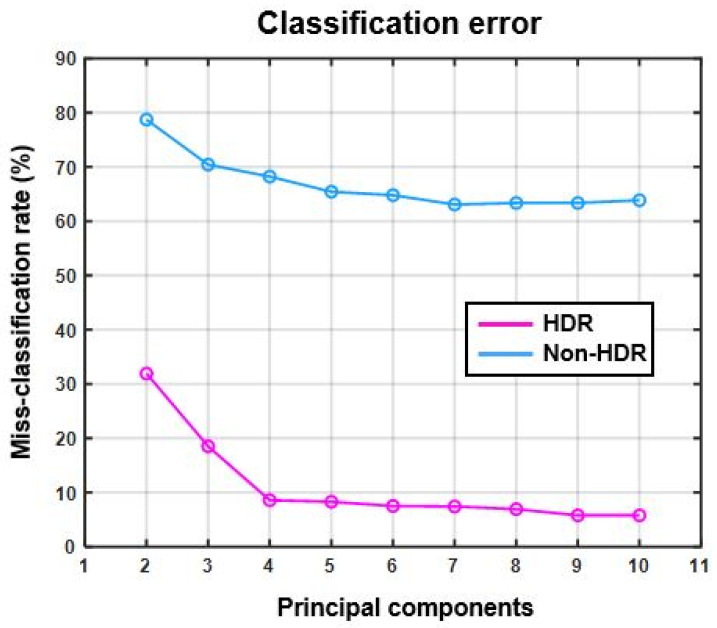
Miss-classification rate (%) vs. number of principal components (test case 2).

Figure 13 shows the misclassification rate for HDR and non-HDR data for the test case 2 where the different recyclable polymers and black plastic were to be classified.

**Figure 14:**
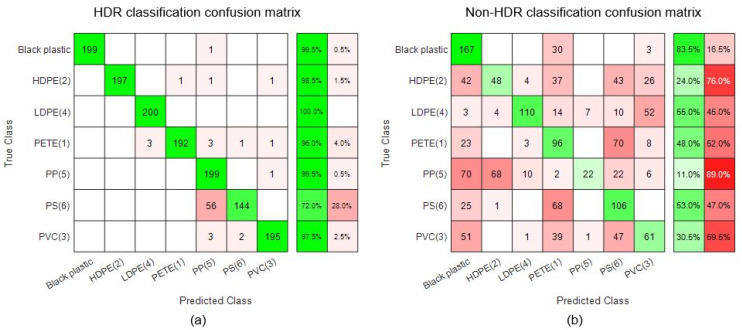
Classification results for test case 2: (**a**) HDR classification confusion matrix, (**b**) nonHDR classification confusion matrix.

In Figure 14, confusion matrices are presented to show the results of classification for the test case 2 where it is possible to see exactly how many vectors of one class are wrongly classified as the members of other classes. Figure 14 also shown the accuracy of classification per each class.

## 4. Discussion

In this study we proposed a multi-exposure method to increase the DR of hyperspectral imaging by line cameras such as the Specim Fx17. The detection of plastic in food waste and polymer classification were used as test case applications, as they present good examples of scenarios in which the DR of a scene can get greater than that of spectral cameras. Throughout this study, special attention was given to black plastic, which is known for its low reflectivity and spectral properties, unlike bright plastics. Black plastics are often made of complex material mixes and are colored black by carbon fillers [26]. These carbon fillings make them difficult to detect in the SWIR region. In Section 2.8 and Section 2.9, the DR of waste samples and the hyperspectral camera are calculated experimentally. Figure 7c and Figure 8 show that the camera does not have enough DR to capture all the meaningful spectral details present in the scene in a single exposure. For polymer classification case the DR of scene was even higher (59 dB). A soft solution was needed to enable the hyperspectral camera to image the scenes with a greater DR than that of the camera itself. In Section 2.10, a multi-exposure method is proposed that can considerably extend the effective DR of hyperspectral imaging by line cameras. In the case of the Specim Fx17, the method allows the camera to meaningfully capture scenes with DR up to 73 dB using multiple exposures, where the camera has an effective DR of approximately 43 dB. This extension was achieved using 27 images taken with exposure times from 1 to 466 ms. With the Specim Fx17 camera, the fastest exposure time is 1 ms. Using this shortest exposure time, it is possible to further extend the effective DR up to 134 dB if needed. A minimum SNR of 20 dB was assumed for the calculations of effective DR. The camera, with its effective DR of 43 dB, was unable to correctly measure the 56 dB and 59 dB range of light intensities generated from reflections in the food waste and polymer samples, respectively. With the extended effective DR using multi exposures, the same method could not only measure the reflections but also provide a saturation margin of 17 dB. The corresponding saturation margin for non-HDR imaging was 3 dB in case 1, a consequence of selecting a single exposure time. To create robustness against large variations in DR from real-world scenarios, a sufficient saturation margin must be provided.

The use of the proposed multi-exposure method also results in better detection and classification of materials present in a scene. In Figure 11a, the non-HDR data of the three classes (food, black plastic, and bright plastic) are more scattered and intermixed than the HDR data of the same classes in Figure 11b. In general, the compact class clusters with small intraclass distances and large interclass distances result in better classification than the comparatively scattered and overlapping clusters.

Figure 12 shows the miss-classification rate as a percentage of two SVM classifiers trained and tested with HDR and non-HDR data. The miss-classification rate of the classifier trained and tested with HDR data is lower than for the classifier trained and tested with non-HDR data. This shows that the use of the multi-exposure method for measuring the spectral reflection with hyperspectral line cameras results in better detection and classification of materials present in a scene with a wider DR than for the hyperspectral camera used.

For test case 1, the HDR and non-HDR data sets were randomly shuffled before being divided into a training set (~80%) and testing set (1200 vectors from all three classes). The testing set was ordered such that the first five hundred vectors were food waste vectors, the second five hundred were bright plastic vectors, and the last two hundred were black plastic vectors. The confusion matrices presented in Table 6**,** show that from the HDR data for any number of principal components used for training, black plastic was never classified as food waste and vice versa. We were able to detect and classify black plastic in food waste with 100% accuracy. This high accuracy was achieved since there was no need to identify the plastics by the type of polymer. Polymer identification from SWIR spectroscopy of black plastics is known to be a difficult case [26]. There were a small number of food waste vectors wrongly classified as bright plastics and vice versa, but overall the miss-classification rate for the HDR data was always lower than for the non-HDR data.

The test application case 2 was included in the final stage of this study with the prime purpose to test the applicability and robustness of the proposed method. The polymer classification results of HDR and non-HDR data are shown in Figure 13 and Figure 14 where the classification success rate of non-HDR data was lower than even 40%. On the other hand, the classification success rate for HDR data was about 95%. The black plastic was treated as a separate.

However, there are shortcomings in this work that limit the empirical evidence for DR being a cause of miss-classification. The black plastic samples contained only one type of black plastic that is used as a container for cooked meals in Swedish superstores. The samples should have included a larger variety of polymers, reflectivity, etc. This study is also limited in size due to its small sample space. However, it becomes more evident that DR is an important consideration for hyperspectral imaging when the mathematical model of DR is analyzed. We argue in this paper that the most common definition of DR is not a satisfactory measure of a hyperspectral camera’s ability to handle large variations of intensity. Instead, we have formulated an effective DR that also includes a requirement for lowest accepted SNR.

Another issue is the high number of exposures (27) used to generate the HDR data in this study. That many exposures of a single line in an industrial application would be very inefficient and very expensive in terms of computation. The number of exposures can be optimized statistically or with the addition of an online algorithm to estimate the minimum required exposures for a given scene. However, optimizing the number of exposures was beyond the scope of this study.

## 5. Conclusions

Many applications of spectral imaging require a spectral camera in SWIR with a high DR to capture all the meaningful spectral details in a scene. However, the technological limitations and high cost of spectral cameras result in them having lower DR than the scenes they are required to capture. In this article, two test application cases were considered where the DR of scenes were higher than the effective DR of spectral camera. Due to its low DR, this camera was unable to accurately measure the spectral reflections of test samples using a single exposure time, especially when the additional requirement of a minimum SNR was added. The concept of minimum SNR was used to define the effective DR. A multi-exposure method is proposed to extend the effective DR of SWIR imaging done by InGaAs-based line cameras. It has been shown that the use of the proposed HDR method reduces the number of classification errors significantly and thus results in much better identification and classification of polymers.

## Figures and Tables

**Table 1:** Description of all samples used in test case 1.

Sample	Description
a–d	The left half of the samples includes general plastic packaging of different colors and types and plastic shopping bags from grocery stores; the right half of the samples includes coarse black plastic.
e–h	The left half of the samples includes general plastic packaging of different colors and types and plastic shopping bags from grocery stores; the right half of the samples includes hand-picked moderately decomposed food items.
i–k	The samples are composed of completely decomposed food waste from which it is not possible to identify any individual food item.

**Table 2:** Types of polymers used in test case 2 samples.

Recycling Number	Abbreviation	Polymer Name
1	PETE or PET	Polyethylene terephthalate
2	HDPE or PE-HD	High-density polyethylene
3	PVC or V	Polyvinyl chloride
4	LDPE or PE-LD	Low-density polyethylene
5	PP	Polypropylene
6	PS	Polystyrene

**Table 3:** Description of polymer samples used in test case 2.

Sample	Description
1	This sample was prepared with all 7 classes of plastics with the aim to attain high DR. The sample contained plastics of different colors.
2–6	These samples were composed of LDPE (4).
7–10	These samples were composed of PVC (3).
11–13	These samples were composed of black plastics.
14–17	These samples were composed of PETE (1).
18–21	These samples were composed of HDPE (2).
22–25	These samples were composed of PP (5).
26–29	These samples were composed of PS (6).

**Table 4:** Number of vectors per data class for HDR and non-HDR datasets (case 1).

Data Class	Training Vectors	Test Vectors	Total
Food	2620	500	3120
Bright plastic	1900	500	2400
Black plastic	1000	200	1200

**Table 5:** Number of vectors per data class for HDR and non-HDR datasets (case 2).

Polymer	Training Vectors	Test Vectors	Total
PETE (1)	2216	200	2416
HDPE (2)	2180	200	2380
PVC (3)	2211	200	2411
LDPE (4)	2191	200	2391
PP (5)	2251	200	2451
PS (6)	2236	200	2436
Black plastic	2091	200	2291

**Table 6:** Confusion matrices for HDR and non-HDR classification (test case 1) from 3 to 5 PC.

3PC Confusion Matrix									
HDR					Non-HDR				
	Actual	Food	Bright Plastic	Black Plastic		Actual	Food	Bright Plastic	Black Plastic
Predict					Predict				
Food		485	9	0	Food		468	41	7
Plastic		15	491	200	Plastic		32	459	193
4PC confusion Matrix									
HDR					non-HDR				
	Actual	Food	Bright Plastic	Black Plastic		Actual	Food	Bright Plastic	Black Plastic
Predict					Predict				
Food		482	6	0	Food		464	37	8
Plastic		18	494	200	Plastic		36	463	192
5PC confusion Matrix									
HDR					non-HDR				
	Actual	Food	Bright Plastic	Black Plastic		Actual	Food	Bright Plastic	Black Plastic
Predict					Predict				
Food		483	10	0	Food		481	12	0
Plastic		17	490	200	Plastic		19	488	200

## Data Availability

Data can be provided on request to corresponding author.
